# The treatment of a Morel-Lavallée lesion of the thigh with incision and drainage along with tissue debridement and a surgically placed drain: A case report and literature review

**DOI:** 10.3389/fsurg.2022.1071421

**Published:** 2023-01-06

**Authors:** Minhua Hu, Junbang Chen, Luyao Ma, Feng Huang, Qunbin Cai

**Affiliations:** ^1^ The First Clinical College, Guangzhou University of Chinese Medicine, Guangzhou, China; ^2^ Department of Orthopedics, The First Affiliated Hospital of Guangzhou University of Chinese Medicine, Guangzhou, China

**Keywords:** Morel-Lavallée lesion, closed degloving injury, post-traumatic subcutaneous cyst, surgical intervention, case report

## Abstract

**Background:**

A Morel-Lavallée lesion (MLL) is a rare closed degloving injury that usually occurs around the hips and is associated with pelvic fractures after high-energy trauma, which is commonly overshadowed by other severe post-traumatic manifestations. An isolated MLL, mostly caused by low-energy violence, is even rarer. Thus, the rates of misdiagnosis and missed diagnosis are often high. In this case report and literature review, we review the pathophysiology, clinical manifestations, imaging data, and treatment of this lesion to increase awareness of this rare disease.

**Case report:**

We report the case of an isolated MLL in the right thigh caused by trauma, which happened to be one of missed diagnosis both at the initial visit and at the return visit of the patient, with a significant sign of a mass on MRI. Given the size of the lesion, open debridement and irrigation were adopted to treat the lesion, and the patient recovered well post-operatively.

**Conclusion:**

Young surgeons should pay attention to the MLL with sufficient recognization to avoid missed diagnosis and misdiagnosis. Comprehensive physical examination and imaging data play important roles in the diagnosis of MLL. In the early stages of this injury, a detailed history review combined with physical examination and MRI, can reduce the rates of missed diagnosis and misdiagnosis. The choice of the therapeutic scheme depends on the size and severity of the lesion. For an isolated MLL, compared with conservative treatments, we suggest that incision and drainage, along with tissue debridement and a surgically placed drain, will reduce the rates of infection and recurrence.

## Introduction

A Morel-Lavallée lesion (MLL) is a post-traumatic closed degloving soft tissue injury that occurs following high-energy trauma by a shearing force, first described by the French surgeon Morel-Lavallée in 1863 (1). The injury frequently occurs in areas of the dense capillary network (e.g., proximal lateral thigh, buttocks, and knee joints) and flexible skin mobility. When a high energy force is imparted to the soft tissue, a shear force can separate the subdermal fat from the superficial fascia, producing a potential space filled with blood, lymph, and necrotic fatty tissue (2). Generally, the MLL is associated with pelvic fractures and hip fractures following high-energy trauma, which is commonly overshadowed by multiple injuries (3). Thus, the rates of misdiagnosis and missed diagnosis are often high. Different from the MLL, an isolated MLL is mainly caused by low-energy force.

This report presents a patient with an isolated MLL without fractures in the right thigh, which happened to be a case of missed diagnosis both at the initial visit and the return visit. Finally, the patient received surgical intervention including incision and drainage along with tissue debridement and a surgically placed drain. We also reviewed the relevant literature and summarized the pathogenesis, clinical manifestations, and treatment strategies for the MLL. Surgeons require an increased understanding of the lesion so that the rates of misdiagnosis and missed diagnosis could be reduced.

## Case report

**Figure 1:**
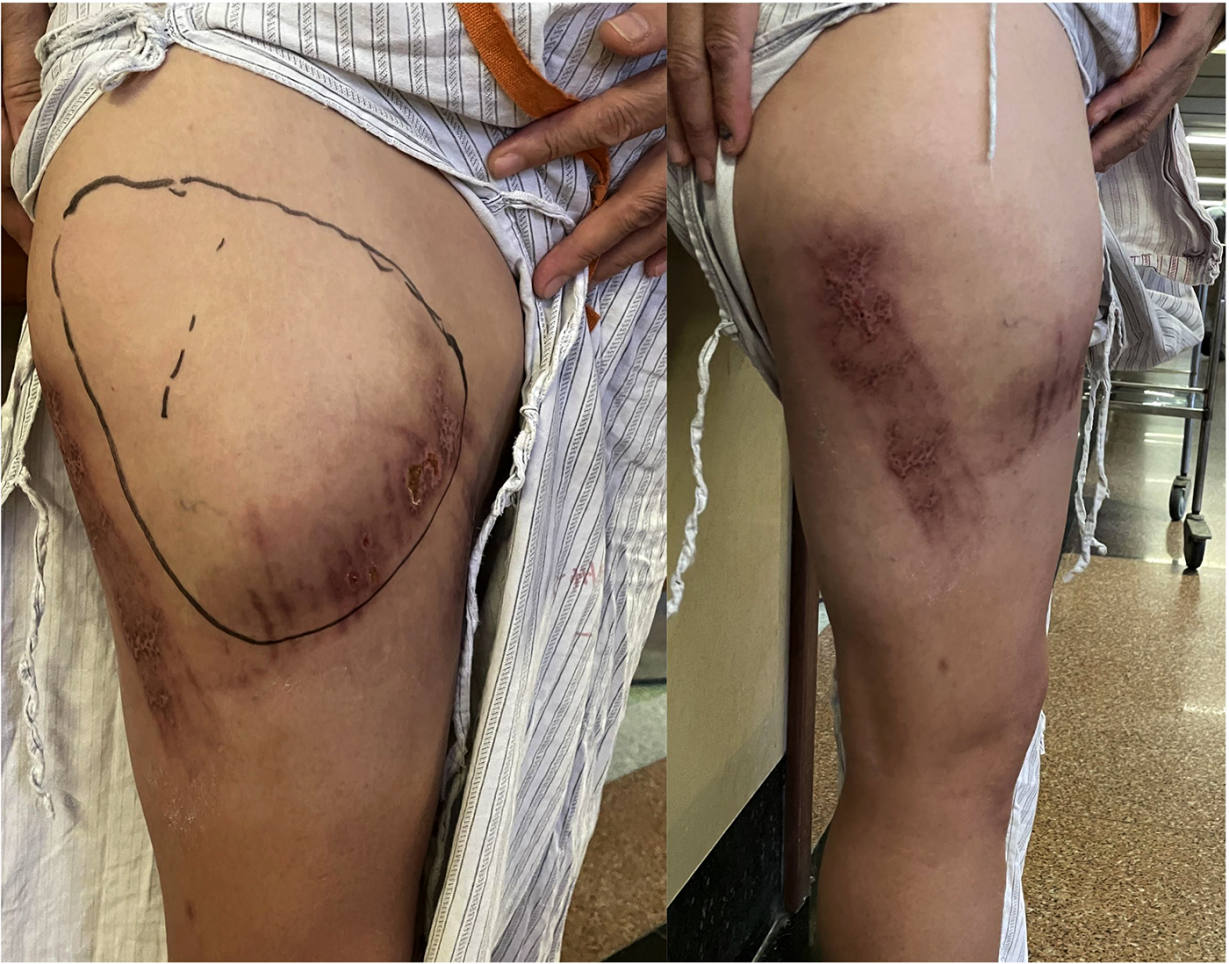
Clinical images. Multiple contusions, bruising, and a wide range of skin fluctuations on the anterolateral right thigh.

**Figure 2:**
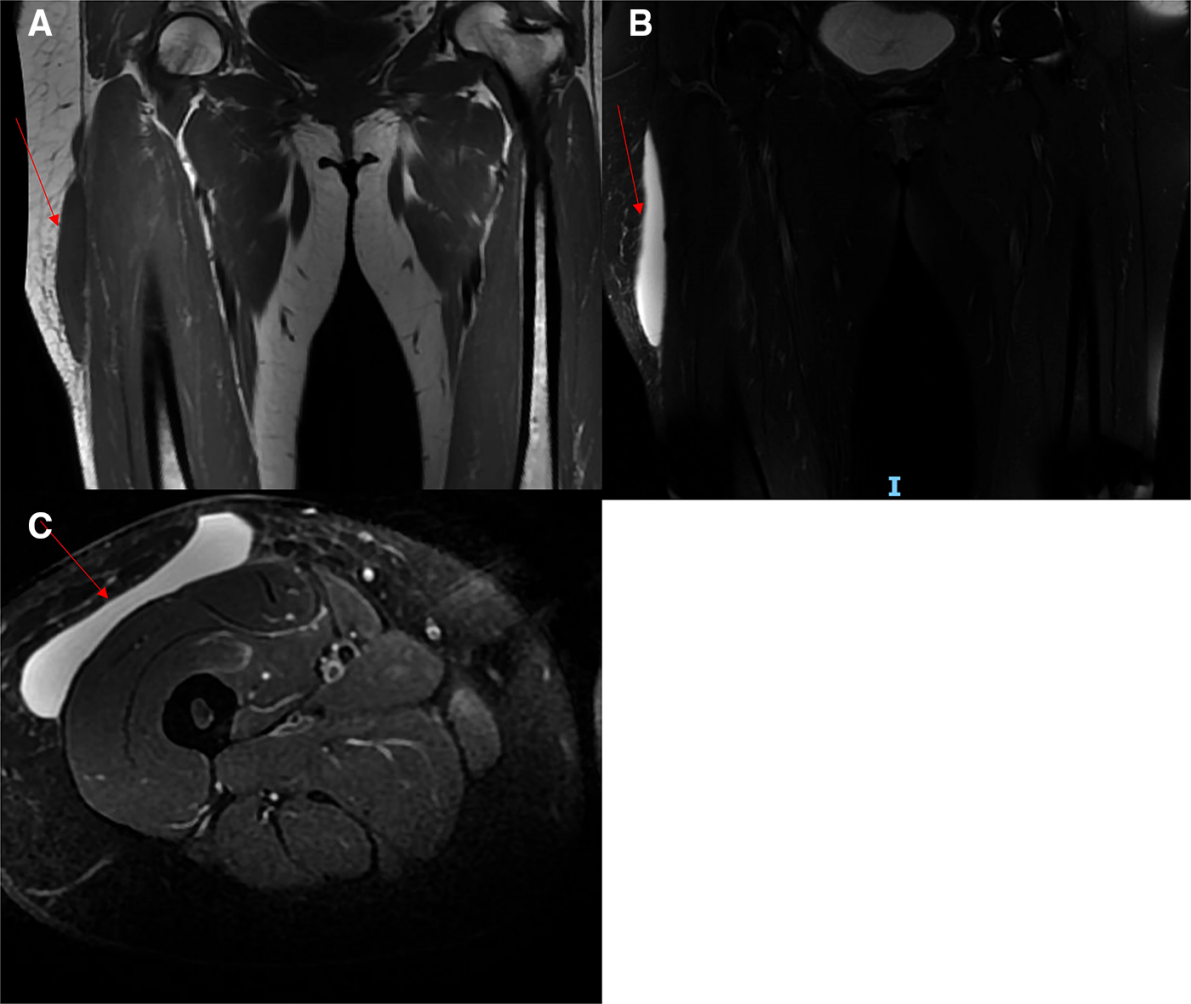
Magnetic resonance imaging of an MLL. (**A**): An abnormal signal intensity was observed in the subcutaneous tissue and the fascia lata of the anterolateral right thigh, which was hypointense on T_1_WI (red arrow). (**B and C**) The lesion was hyperintense on T_2_WI (red arrow).

A 49-year-old healthy female patient presented with anterolateral pain in the upper right thigh, multiple skin contusions, and pain in the left wrist and chest after falling from an electric bike on August 10, 2022. The contusion of the skin of the right thigh was taken into account, and the wound was disinfected with iodophor after the fracture was excluded from the community hospital. However, pain at the lesion site increased, resulting in redness and ecchymosis. When the patient was referred to the outpatient department upon her return visit, an MRI of the right thigh was performed, which suggested right thigh anterolateral subcutaneous effusion and soft tissue mild edema. However, soft tissue edema was diagnosed, and an inexperienced surgeon provided a conservative treatment by administering oral analgesics. Suffering from long-term unremitted pain and swelling, the patient was admitted to the inpatient department for further treatment three weeks later. The symptoms after admission were mainly swelling and pain in the right thigh with anterior and lateral skin abrasions and a slightly red complexion but normal skin temperature. A wide range of skin fluctuations and tenderness existed in the anterolateral thigh, but there was no vertical axis pain in the right lower limb (Figure 1). Magnetic resonance imaging showed no fracture, but an abnormal signal was located in the anterolateral part of the right thigh, between the iliac fascia and the subcutaneous soft tissue. The boundary of the subcutaneous mass was clear, with a range measuring 107  ×  15  ×  150 mm, and no pseudo fibrous capsule was found. Furthermore, the lesion was hypointense on T1-weighted images (T_1_WI) (Figure 2A) and hyperintense on T2-weighted sequences (T_2_WI) (Figures 2B,C). The lesion's clinical manifestations and imaging appearances were consistent with an MLL, therefore, we diagnosed it as a closed degloving soft tissue injury.

**Figure 3:**
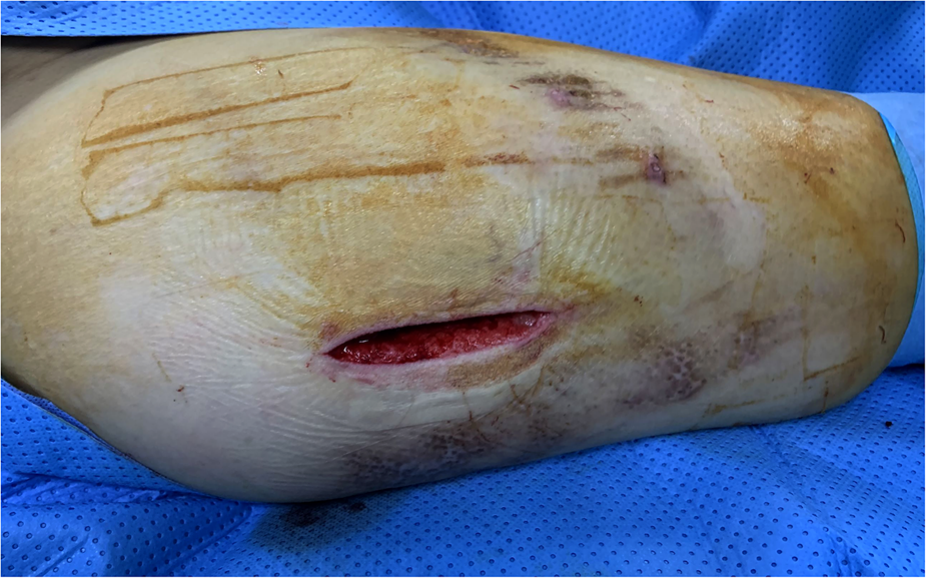
Operation data. No skin fluctuations and no cavity were observed between the subcutaneous fat and the fascia iliaca of the anterolateral right thigh.

**Figure 4:**
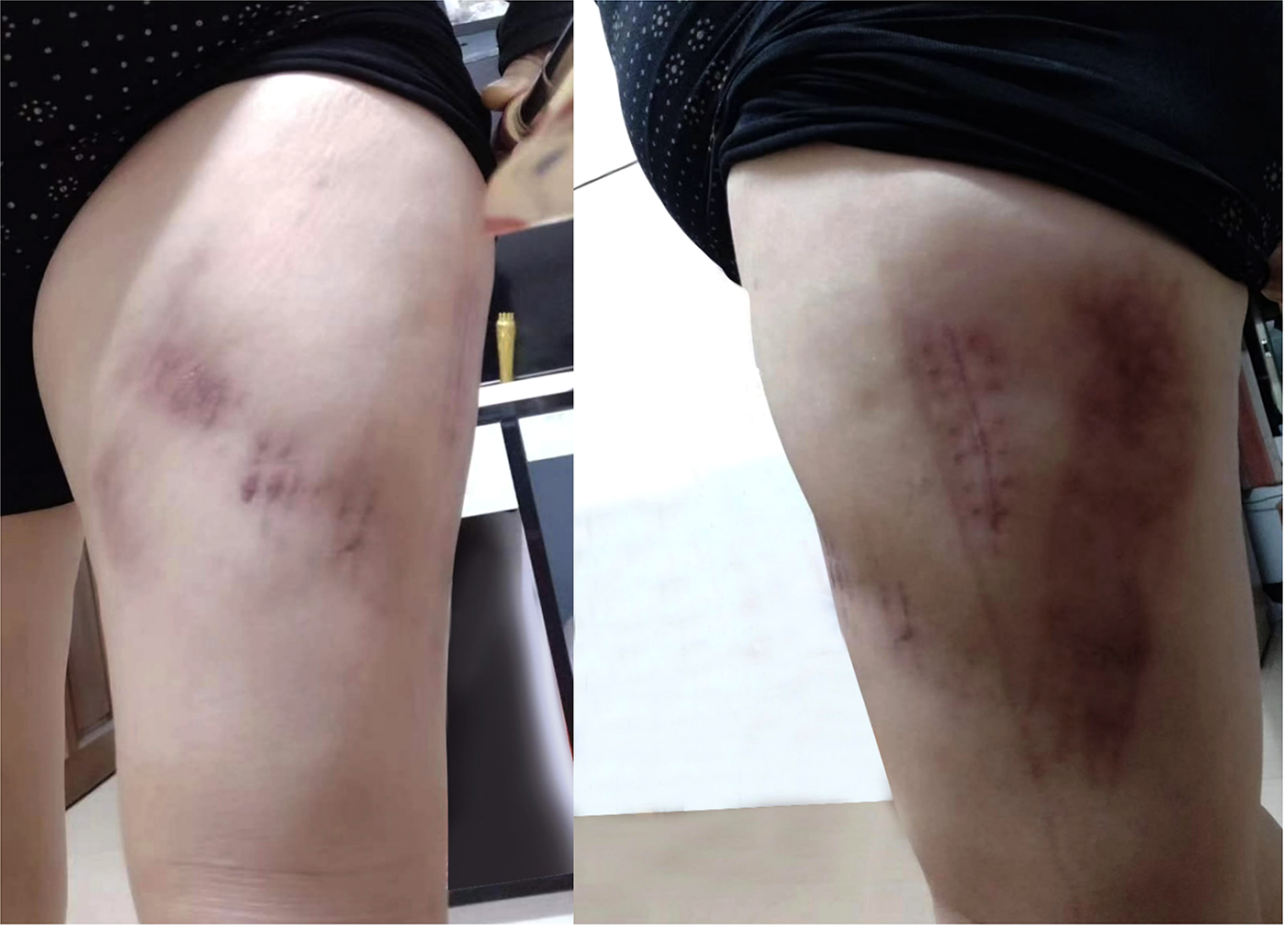
Clinical images after healing. Pain, swelling, and previous skin fluctuations in the patient’s right leg disappeared.

Given the size of the lesion, the patient underwent surgical intervention which included an incision and drainage along with tissue debridement and a surgically placed drain. After completion of the sterile preoperative preparation, an incision in the skin and subcutaneous tissue was made along the anterolateral side of the right thigh, with profuse exudation of light red fluids. We found a space between the fascia lata and the subcutaneous fat, which should not exist in a standard anatomical structure. After irrigating the cavity with a large amount of iodophor, hydrogen peroxide, and saline, we used vacuum-assisted closure therapy to promote cavity closure. The cavity was filled with polyethylene alcohol–hydrated seaweed salt foam dressing, with a semi-permeable bio-membrane covering the incision. Then, a continuous vacuum sealing drainage (VSD) device and a percutaneous drain were used to exhaust blood, lymph, and fatty debris. After 1 week, we removed the VSD and used non-absorbable, non-braided sutures to close the deep fascia and skin of the incision (Figure 3), with an elastic bandage binding the injury area of the right thigh. Finally, the patient reported favorable outcomes; wound pain and a wave-like feeling in the anterolateral right thigh disappeared at discharge (Figure 4).

## Discussion

The characteristics of this case report are consistent with those of a Morel-Lavallée lesion, including the injury mechanism, clinical manifestations, and imaging data. Generally, the lesion is caused by a shearing force, separating the subcutaneous fat and deep fascia and creating a space between them. At the same time, capillaries and lymphatics in soft tissue become disrupted with shearing injury, leading to an accumulation of lymph, blood, debris, and fat in the interfacial plane. After this, necrosis of fat liquefaction occurs in the lacunae, and these components are gradually absorbed to form a serum fluid wrapped in hemosiderin layer, leading to local inflammation and pseudo capsule formation (2). Due to these pathological features, infection and recurrence rates are often high. Bacterial colonization has been reported in nearly half of Morel-Lavallée lesion samples, which poses a great challenge for treatment and prognosis (4).

The clinical presentations of an isolated MLL without fractures are non-specific and include pain, ecchymosis, swelling, and fluctuating sensations in the injured area (5). In the early stages of injury, the MLL is not easily distinguished from soft tissue injury. Pain and swelling are the most frequent clinical complaints at this stage, and skin hypermobility can be found in palpation due to the separation of subcutaneous tissue and deep fascia. However, skin hypermobility does not appear until lymph, blood, debris, and fat in the interfacial plane penetrate the cavity. In addition, the patient may perceive progressive aggravation of local pain and swelling, with a reddened skin color and increasing skin temperature. When pseudo capsules are formed, the local skin of the lesion will be rough and rugged. If not handled in time or handled improperly, infection and necrosis may occur in the local skin and subcutaneous tissue (6, 7). Therefore, the lesion is often not recognized, resulting in missed diagnosis at the patients’ first visit. Due to a lack of awareness of this injury, inexperienced surgeons may consider it a simple soft tissue injury after completing a series of physical examinations and preliminary imaging studies. Moreover, when more severe injuries such as pelvic fractures and intertrochanteric fractures co-occur, inexperienced surgeons may focus on fractures and ignore this injury between the superficial and the deep fascia. As reported in this study, the isolated MLL showed atypical symptoms similar to simple skin contusion at our patient’s first visit, thus the skin contusion in the right thigh was examined at a community hospital. Although the MRI suggested imaging features typical of subcutaneous effusion, an inexperienced surgeon still misdiagnosed this as an isolated MLL.

Generally, it is not easy to diagnose an MLL, and therefore, a review of the clinical history, a comprehensive physical examination, and advanced imaging modalities are highly important. Ultrasound (US), computed tomography (CT), and magnetic resonance imaging (MRI) can be used to provide additional information. US demonstrates heterogeneous echogenicity, appearing as a fluid collection in the area of the lesion. This variation is often not specific, and a simple hematoma, effusion, abscess, and tumor can show similar characteristics to this lesion, making it difficult to determine the nature of heterogeneous echogenicity. However, their imperceptible specific appearance in the acute lesion (e.g., lobular appearance, adjacent disruption of fascial planes, internal echogenic debris, and fat globules) is more likely to be observed using a high-frequency linear probe in US (8–10). Typically, CT has been the initial modality of choice in an emergency, which can reveal low density fat-fluid levels, fluid-fluid levels, or blood-fluid levels. Due to the presence of lymph in the cavity, the CT density of this lesion is usually lower than that of simple hematomas (11). MRI has high sensitivity and specificity and is considered the “golden criterion” for diagnosing an MLL. Based on the different characteristics of MRI, six types of MLL were described by Melado and Bnecardino, with seroma, subacute hematoma, and chronic organizing hematoma the commonest in clinical practice (12). In general, seroma is hypointense on T_1_WI and hyperintense on T_2_WI, and occurs in acute and chronic lesions without forming a fibrous capsule. In comparison, subacute hematoma is hyperintense both on T_1_WI and on T_2_WI due to the presence of methemoglobin. Internal inhomogeneity can be observed on MRI, with the presence of fat globules and internal septations. Furthermore, variations in the MLL tend to be more pronounced after fibrous capsule formation. Chronic-organizing hematoma is hypointense or intermediate-intense on T_1_WI and heterogeneous intermediate–intense on T_2_WI, with the formation of capsule. As different contents of lesions such as hemosiderin granulation tissue, necrotic debris, fibrin, and blood clots exist in the cavity, different signals will be seen on MRI (10). According to the classification proposed by Melado and Bnecardino, the isolated MLL that we report here is classified as a type I seroma. Despite the lack of US and CT image data, the MRI is typical, which is consistent with the MRI of an MLL, showing a spindle-shaped mass between the anterolateral subcutaneous tissue of the right thigh and the fascia lata, with well-defined margins, hypointensity on T_1_WI, and hyperintensity on T_2_WI. Moreover, these are frequently seen in the early stage of the lesion but sometimes can be found in the chronic phase. The morphology of liquid accumulation usually shows a laminar on MRI and is primarily non-capsulated (13).

However, there is no guideline or consensus on the treatment of an MML, despite many MLL reported treatments. Conservative management options include compression bandaging, percutaneous puncture aspiration, and sclerosants, either provided separately or combined together. In clinical practice, the management of an MLL is highly variable, and therapeutic scheme choice depends on the size and severity of the lesion. Local compression bandaging is considered a simple but effective therapy for minor lesions and is frequently coordinated with adjuvant therapy such as physical therapy, bed rest, and oral NSAIDs. A retrospective review showed that all 25 adolescents with an MLL, treated with compression bandaging, yielded favorable outcomes (14). Nevertheless, outcomes may be limited when the lesion occurs in the greater trochanter or other specific body regions. Furthermore, it is difficult for a patient under compression bandaging to rapidly recover, and usually the road to recovery is longer (15). Percutaneous puncture aspiration is also used for treating a minor acute lesion, and it is easier to operate and obtain successful results using US. However, high infection and recurrence rates limit the application of percutaneous puncture aspiration. If there is aspiration of more than 50 ml, the rate of recurrence is high at 80% (16). Sclerotherapy is considered a practical option for seroma treatment. The administration of sclerosing agents can effectively reduce the recurrence rate by injecting doxycycline and tetracycline into the cavity to induce the formation of fibrosis and a closed, dead cavity; this technique is often applied in conjunction with percutaneous puncture aspiration (17). However, surgeons should be alert to skin necrosis and infection when the patient is treated with sclerosing agents (18). Surgical interventions include incision and drainage, tissue debridement, vacuum sealing drainage, percutaneous quilting, and endoscopic debridement, which can be used for larger lesions or lesions with underlying fractures. Recently, minimally invasive surgery has advanced the treatment of MLLs, resulting in fewer surgical scars and lower infection rates. Percutaneous quilting has been proven as an effective therapy for reducing the recurrence rate by eliminating the dead space between the subcutaneous tissue and the deep fascia, and is frequently combined with other operations. Kumar et al. treated 22 MLL patients with a percutaneous quilting technique without adverse events. In this study, the skin and deep fascia were tightly sutured with heavy, non-absorbable, non-braided sutures, and a suction drain tip was used as a marker to ensure that the sutures engaged the deep fascia to the skin (19). Li et al. demonstrated a minimally invasive incision and a nose ring drainage technique for treating lower limb MLLs. All patients recovered without complications and an excellent cosmetic effect was obtained (20). The endoscopic technique is also recommended as a minimally invasive therapy, which provides a distinct internal view of the lesion, allowing removal of serous effusion, necrotic adipose tissue, blood, and false bursa in the cavity. In clinical practice, surgeons often combine endoscopy and other operations to treat an MLL. Liu et al. presented a study in which endoscopic debridement was used and combined with percutaneous cutaneous-fascial suture to treat eight patients (21). Baris et al. treated a chronic MLL in the knee of a professional soccer player using endoscopic debridement and fibrin glue injection after conservative management failed (22). However, MLL patients who have other concomitant injuries may require additional management, which depends on the condition of skin necrosis and the severity of the injury. A recent study described a novel technique to treat pelvic fracture with a severe MLL. After the pelvic fractures of eight patients were fixed with channel screws, the skin in the MLL area was debrided and excised, and skin stretch technology was used to promote wound healing (23). Sigrid et al. reported the case of a patient on whom a veraflo vac dressing was used to treat MLL wounds with friction burn, which prevented excessive debridement and infection in the patient (24).

In this report, surgical interventions were adopted to treat an isolated MLL. The patient had been misdiagnosed, and was admitted to the hospital for treatment after oral analgesics and rest proved ineffective. Because of the extensive range of skin and high liquid accumulation, it was not suitable to apply a compression bandage. Although percutaneous puncture drainage can remove effusion in the cavity, a high risk of recurrence and infection may exist due to multiple skin contusions in the right thigh. Furthermore, when we reviewed the patient's imaging data, no fibrous capsule was visible in MRI. Given the above, we opted for performing incision and drainage, along with tissue debridement and a surgically placed drain to clean the content of the cavity. Elastic bandage compression was used to close the cavity. As a routine therapy, incision and drainage, along with tissue debridement, is successful in adequately debriding necrotic components and creating good preoperative conditions for other injuries. Interestingly, compared with compression bandaging or percutaneous puncture aspiration, incision debridement and drainage have minimal recurrence rates (16). Moreover, this approach is simple; although open drainage and debridement is associated with a risk of expanding trauma compared with endoscopic debridement or percutaneous quilting. It does not require advanced equipment and can be performed even in poorly equipped hospitals, and even by young surgeons. In order to promote closure of the cavity, a vacuum sealing drainage device was used to remove the necrotic tissue after debridement. Currently, vacuum-assisted closure therapy is frequently applied for treating MLLs (25). It effectively promotes granulation tissue growth, dead space obliteration, and healthy tissue restoration, and it reduces the risk of infection. A recent study on 15 MLL patients described this modality. After incision debridement combined with vacuum-assisted closure was performed, all patients reported favorable outcomes with no evidence of lesion recurrence or infection, thus proving its effectiveness (26).

## Conclusion

In summary, young surgeons should pay attention to an MLL, with sufficient recognization to avoid both missed diagnoses and misdiagnoses. Comprehensive physical examination and imaging data play essential roles in diagnosing an MLL. In the early stages of this injury, a detailed history review combined with physical examination and MRI can reduce the rates of missed diagnosis and misdiagnosis. The choice of the therapeutic scheme depends on the size and severity of the lesion. For an isolated MLL, compared with conservative treatments, we suggest that incision and drainage, along with tissue debridement and a surgically placed drain, will reduce infection and recurrence rates.

## Data Availability

The original contributions presented in the study are included in the article/Supplementary Material, further inquiries can be directed to the corresponding author/s.

## References

[1] Kumar Y Hooda K Lo L Karol I. Morel-Lavallée lesion: a case of an American football injury. Conn Med. (2015) 79:477–8. PMID: 26506679

[2] Scolaro JA Chao T Zamorano DP. The Morel-Lavallée lesion: diagnosis and management. J Am Acad Orthop Surg. (2016) 24:667–72. 10.5435/JAAOS-D-15-00181 27579812

[3] Hakim S Ahmed K El-Menyar A Jabbour G Peralta R Nabir S Patterns and management of degloving injuries: a single national level 1 trauma center experience. World J Emerg Surg. (2016) 11:35. 10.1186/s13017-016-0093-2 27468300 PMC4962500

[4] Hak DJ Olson SA Matta JM. Diagnosis and management of closed internal degloving injuries associated with pelvic and acetabular fractures: the Morel-Lavallée lesion. J Trauma. (1997) 42:1046–51. 10.1097/00005373-199706000-00010 9210539

[5] Pothiawala S Miranda R Civil I. Not all post-traumatic swellings are haematomas: be alert to a Morel-Lavallée lesion. Lancet. (2022) 400(10345):e1. 10.1016/S0140-6736(22)01058-3 35780796

[6] Luisetto M Legrand A Vandromme E Boulares S Delahaut O. Morel-Lavallée lesion associated with atypical skin damage: a case report. Acta Orthop Belg. (2021) 87:751–4. 10.52628/87.4.21 35172443

[7] Nakajima T Tada K Nakada M Matsuta M Tsuchiya H. Two cases of Morel-Lavallée lesion which resulted in a wide skin necrosis from a small laceration. Case Rep Orthop. (2020) 2020:5292937. 10.1155/2020/5292937 32257483 PMC7106902

[8] Tulip SL Rao RR Sielaff A Theyyunni N Burkhardt J. Ultrasound utility in the diagnosis of a Morel-Lavallée lesion. Case Rep Emerg Med. (2017) 2017:3967587. 10.1155/2017/3967587 28255470 PMC5309421

[9] McLean K Popovic S. Morel-Lavallée lesion: AIRP best cases in radiologic-pathologic correlation. Radiographics. (2017) 37:190–6. 10.1148/rg.2017160169 28076017

[10] Coninck TD Vanhoenacker F Verstraete K. Imaging features of Morel-Lavallée lesions. J Belg Soc Radiol. (2017) 101:15. 10.5334/jbr-btr.1401 30498807 PMC6251078

[11] McKenzie GA Niederhauser BD Collins MS Howe BM. CT Characteristics of Morel-Lavallée lesions: an under-recognized but significant finding in acute trauma imaging. Skeletal Radiol. (2016) 45:1053–60. 10.1007/s00256-016-2374-y 27098352

[12] Mellado JM Bencardino JT. Morel-Lavallée lesion: review with emphasis on MR imaging. Magn Reson Imaging Clin N Am. (2005) 13:775–82. 10.1016/j.mric.2005.08.006 16275583

[13] Volavc TS Rupreht M. MRI of the Morel-Lavallée lesion – a case series. Radiol Oncol. (2021) 55:268–73. 10.2478/raon-2021-0018 33792213 PMC8366731

[14] Kushare I Ghanta RB Wunderlich NA. Morel-Lavallée lesions (internal degloving injuries) of the lower extremity in the pediatric and adolescent population. Phys Sportsme. (2021) 49:182–6. 10.1080/00913847.2020.1803712 32735762

[15] Rodríguez-Roiz JM Burillo JM Díaz JSS. Morel-Lavallee lesions. Size matters? Treatment and time of disability. Injury. (2022) S0020-1383:00794-X. 10.1016/j.injury.2022.10.023 36328805

[16] Nickerson TP Zielinski MD Jenkins DH Schiller HJ. The mayo clinic experience with Morel-Lavallée lesions: establishment of a practice management guideline. J Trauma Acute Care Surg. (2014) 76:493–7. 10.1097/TA.0000000000000111 24458056

[17] Gupta A Kumar V Agarwal A Suresh A. Management of recurrent post-traumatic seroma of thigh (Morel-Lavallée lesion) by percutaneous aspiration and sclerotherapy using tetracyclines (PAST). BMJ Case Rep. (2021) 14:e238802. 10.1136/bcr-2020-238804 PMC7813355 33462032

[18] Sood A Kotamarti VS Therattil PJ Lee ES. Sclerotherapy for the management of seromas: a systematic review. Eplasty. (2017) 17:e25. PMID: 28890747 PMC5575675

[19] Kumar G Pandiyan A Theruvil B. Percutaneous quilting technique for the treatment of Morel-Lavallée lesion. Indian J Orthop. (2020) 54:580–6. 10.1007/s43465-020-00097-4 32850020 PMC7429629

[20] Li P Ning XH Jia L Du GQ Jiang SY Gong ZH A minimally invasive incision and loop drainage technique for the treatment of lower limb Morel-Lavallée lesions: nose ring drainage technique. Injury. (2020) 51:570–3. 10.1016/j.injury.2019.12.014 31852590

[21] Koc BB Somorjai N Kiesouw EPM Vanderdood K Meesters-Caberg M Draijer FW Endoscopic debridement and fibrin glue injection of a chronic Morel-Lavallée lesion of the knee in a professional soccer player: a case report and literature review. Knee. (2017) 24:144–8. 10.1016/j.knee.2016.10.017 27887784

[22] Gehring MB Yang KJ Cusic JG Ramamurthi A Matloub HS Argenta AE. A novel surgical technique for resuspension of digital pulp tissue after degloving injuries. Plast Reconstr Surg Glob Open. (2019) 7:e2600. 10.1097/GOX.0000000000002600 32537317 PMC7288873

[23] Huang W Luo YM Yang EP. Application of channel screw combined with skin-stretching technique in treatment of pelvic fracture with severe Morel-Lavallée lesion. Zhongguo Xiu Fu Chong Jian Wai Ke Za Zhi. (2021) 35:973–7. 10.7507/1002-1892.202103111 34387424 PMC8403993

[24] Blome-Eberwein SA. Morel-Lavallée lesion with friction burn: management using veraflo vac dressing, preserving body contour. Plast Reconstr Surg Glob Open. (2020) 8:e2747. 10.1097/GOX.0000000000002747 32440417 PMC7209856

[25] Malagelada F König TC Bates P. Combination of a drainage tube and fenestrated topical negative-pressure device for the management of Morel-Lavallée lesions. Ann R Coll Surg Engl. (2016) 98:341–2. 10.1308/rcsann.2016.0118 27087331 PMC5227036

[26] Marangi GF Segreto F Coppola MM Arcari L Gratteri M Persichetti P. Management of chronic seromas: a novel surgical approach with the use of vacuum assisted closure therapy. Int Wound J. (2020) 17:1153–8. 10.1111/iwj.13447 32716145 PMC7948681

